# Supplementary material for: Allosteric Inhibition of c-Abl to Induce Unfolded Protein Response and Cell Death in Multiple Myeloma
Source: Int J Mol Sci. 2022 Dec 18;23(24):16162. doi: 10.3390/ijms232416162 (PMC9786043; doi:10.3390/ijms232416162)
Supplement: Supplementary file 1 [file ijms-23-16162-s001.zip › ijms-2002935-supplementary.pdf]

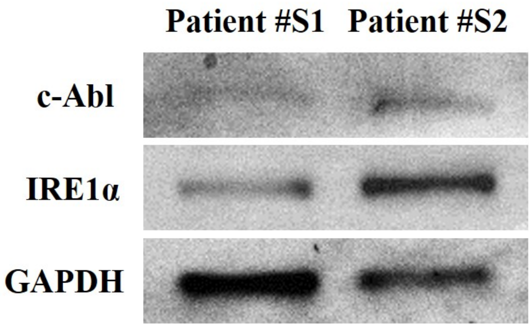

Figure S1. Whole-cell lysates of primary patient-derived myeloma cells were subjected to Western blotting using antibodies against c-Abl, IRE1α, and GAPDH.

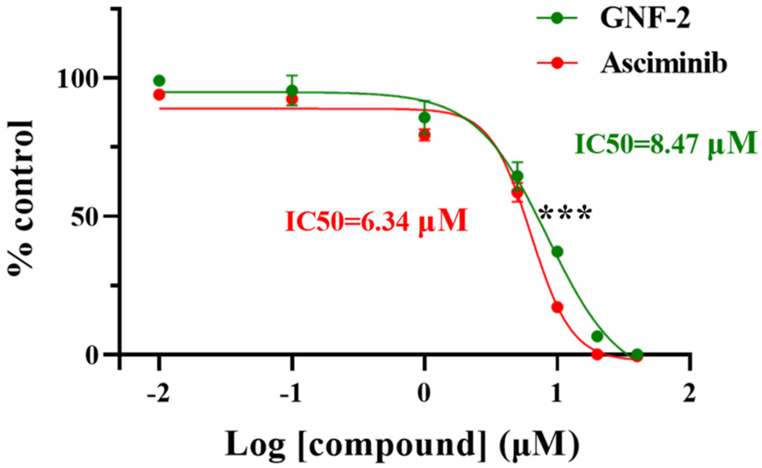

Figure S2. KMS-12-PE cells were treated with GNF-2 (0-40 µM) or asciminib (0-40 µM) for 72 h. In each case, the cell viability of triplicate cultures was assessed using the Cell Counting Kit-8 (CCK-8) and is expressed as the percentage of the value obtained for the untreated control. Data were analyzed with two-way analysis of variance, followed by post-hoc Sidak's multiple comparison test; ***$p<0.001$.

Table S2. Clinical characteristics of patients with multiple myeloma in this study

Case	Sex	Age at diagnosis (years)	NCC (x10^4/µL) in BM[1]	Myeloma % in BM	Serum M protein type
MM-1	F	81	5.15	16.8	IgGκ
MM-2	M	79	5.5	8.4	IgAκ
MM-3	M	80	23.7	49.2	IgGκ
MM-4	M	83	10.6	8.0	IgAλ
MM-5	M	73	4.2	29.6	IgGκ
MM-6	F	72	3.5	27.6	BJPλ

BM: bone marrow, F: Female, M: Male, MM: Multiple Myeloma, NCC: Nucleated Cell Count, BJP: Bence Jones Protein.

[1]Normal range of NCC in the BM is 10-25 x10^4/µL

Table S3. Quantitative **RT-PCR primer sequences used in this study**

Gene	Forwards 5'→3'	Reverse 5'→3'
ACTB	AGAGCTACGAGCTGCCTGAC	AGCACTGTGTTGGCGTACAG
CHOP	AGAGCTACGAGCTGCCTGAC	AGCACTGTGTTGGCGTACAG
sXBP1	GAGTCCGCAGCAGGTG	TCCTTCTGGGTA GACCTCTGGGAG
ATF4	AAGGCGGGCTCCTCCGAATGG	CAATCTGTCCCGGAGAAGGCATCC
ATF6	ACCTGCTGTTACCAGCTACCACCCA	GCATCATCACTTCGTAGTCCTGCCC
